# PB.41. What is the incidence of internal mammary node lymphadenopathy on CT in primary breast cancer patients within 1 year of a diagnosis?

**DOI:** 10.1186/bcr3707

**Published:** 2014-11-03

**Authors:** S Savaridas, J Cox

**Affiliations:** 1 University Hospital of North Durham, Durham, UK

## Introduction

The importance of **internal mammary nodes (IMNs) **in the staging and treatment of breast cancer patients is controversial. Lymphoscintigraphic studies have demonstrated that a significant proportion of breast cancers have primary or partial IMN drainage. However, historical studies demonstrated no overall benefit of treating IMNs with either surgery or radiotherapy. But with recent advances in treatment, targeted radiotherapy of the IMN chain is now possible. Thus we sought to establish the frequency of IMNs within our primary breast cancer population.

## Methods

Retrospective cohort study of all spiral CT thorax performed on patients within 12 months following the primary diagnosis of breast cancer from January 2009 to December 2012. The number and size of any IMNs were recorded.

## Results

A total of 830 patients were diagnosed with primary breast cancer within our time frame; 150 patients were included in this study. Of these, 40% (*n *= 60) had IMNs present, although the majority were small (<5 mm). However, 16% (*n *= 24) had larger nodes >5 mm present on CT.

## Conclusion

We have demonstrated that IMNs are present in a substantial number of our primary breast cancer patients. We suggest that routine imaging of the IMN chain as well as axilla should be considered in the staging of breast cancer.

